# Vigorous physical activity is important in maintaining a favourable health trajectory in active children: the CHAMPS Study-DK

**DOI:** 10.1038/s41598-021-98731-0

**Published:** 2021-09-28

**Authors:** Martin Sénéchal, Jeffrey J. Hebert, Timothy J. Fairchild, Niels Christian Møller, Heidi Klakk, Niels Wedderkopp

**Affiliations:** 1 Faculty of Kinesiology, University of New Brunswick, New Brunswick, Canada; 2 Cardiometabolic Exercise & Lifestyle Laboratory, Faculty of Kinesiology, University of New Brunswick, New Brunswick, Canada; 3 The Centre for Molecular Medicine and Innovative Therapeutics, Murdoch University, Murdoch, Australia; 4 Department of Sports Science and Clinical Biomechanics, Research Unit for Exercise Epidemiology, Center for Research in Childhood Health, Faculty of Health Science, University of Southern Denmark, Odense, Denmark; 5 Center for Applied Health Science, University College South (UC SYD), Haderslev, Denmark; 6 The Orthopedic Department, University Hospital of South West Jutland, Esbjerg, Denmark; 7 Department of Regional Health Research, University of Southern Denmark, Odense, Denmark

**Keywords:** Physiology, Health care, Risk factors

## Abstract

Physical activity (PA) is critical to improving health factors such as weight, adiposity, and aerobic fitness. However, children who meet PA guideline recommendations demonstrate developmental differences in health-related outcomes. To investigate prospective associations between PA behaviour (overall PA and PA intensity) and trajectories of health-related factors among physically active children. This prospective study (2.5 years) included 391 children (baseline age: 8.1 ± 1.4 years; girls 36.3%) from ten public schools. All children performed 60-min or more of moderate-to-vigorous physical activity (MVPA) per day objectively measured. Trajectories of BMI, waist circumference, and aerobic fitness were constructed with a group-based multi-trajectory model. Three trajectory subgroups were identified: ‘high fitness/normal weight’ (48.4% of children), ‘moderate fitness /normal weight’ (42.5% of children), and ‘low fitness/overweight-obese’ (9.1% of children). Children performing higher overall PA, were less likely of being classified as members of the ‘Low Fitness/Overweight-Obese’ [Relative Risk Ratio (RRR and 95% CI) = 0.56 (0.37 to 0.85) compared to ‘high fitness/normal weight’ subgroup. Each additional 5% in light PA time was associated with approximately twofold [RRR 2.12 (1.24–3.61)] increased risk of being in the ‘low fitness/overweight-obese’ trajectory relative to the ‘high fitness/normal weight’ trajectory. Each additional 2% in vigorous-PA time was associated with a 42% and 85% reduced risk (relative to ‘high fitness/normal weight’) of being in the ‘moderate fitness/normal weight’ [RRR 0.58 (0.38–0.96)] and ‘low fitness/overweight-obesity’ [RRR 0.15 (0.06–0.37)] trajectory, respectively. Overall PA and additional time in vigorous-PA was associated with improved health-related outcomes, while light PA was negatively associated with health-related outcomes among children who adhere to PA guideline recommendations. Vigorous PA was the strongest predictor of the health trajectories. All PA guidelines for children should place greater emphasis on the importance of vigorous PA.

## Introduction

Insufficient physical activity (PA) is the fourth leading cause of global mortality, representing about 6% of premature deaths^1^. In 2013, physical inactivity was estimated to cost the global health-care system $53.8 billion^2^. Improving PA behaviour is therefore a cornerstone strategy to prevent, manage, and treat many chronic diseases^3^. Even from a young age, high levels of PA are associated with improved cardiorespiratory fitness and reduced cardiometabolic risk^4–6^. Consequently, health guidelines recommend that young people engage in at least 60 min of moderate-to-vigorous PA (MVPA) per day^7^. Yet, less than half of children between 6 and 19 years meet health-related PA guideline recommendations^8–10^.

In addition to the challenge posed by the relatively low number of individuals meeting health-related PA guidelines, there is large heterogeneity in response to PA in both adults and children. For example, the average increase in aerobic fitness following a 24-week aerobic exercise programme was approximately 25%, however individual responses ranged from 0 to 100% in adults^11,12^. Work from our group^13,14^ and others^15,16^ has demonstrated similar heterogeneous responses in young people at risk of chronic disease. The variation in PA response presents a challenge from a public health standpoint, as health-related PA guidelines focussing on time in MVPA are unlikely to be efficient for all children^17^.

Considering the strong and consistent associations between low fitness, elevated waist circumference and high BMI in childhood with increased health risk factors^18^, these measures are often used as health-related outcomes in children^19^. A recent study reported that one in 10 children who met PA guideline recommendations follow unfavorable health trajectories characterised by overweight/obesity and low aerobic fitness^20^. This suggests that PA guideline recommendations may be insufficient to mitigate health risk factors in some children. Identifying modifiable factors including PA characteristics that predict health outcomes, would benefit health-related PA prescription. Therefore, this study aimed to investigate the prospective association between PA behaviour (overall PA and PA intensity) and health-related trajectories of aerobic fitness, waist circumference, and BMI in children meeting health-related physical activity guidelines.

## Methods

### Study design and participants

Data from the Childhood Health, Active, and Motor Performance School Study Denmark (CHAMPS Study-DK) were used. The study sample and procedures of this quasi-experimental study have been published elsewhere^21,22^. Between 2008 and 2011, 10 public schools participated in the study. The current analysis was restricted to (1) participants who performed a minimum of 60 min of daily MVPA at 12 months and 22 months, which represents 43.2% of the original sample, and (2) participants who have a minimum of three time point measurements for health trajectories variables (cardiorespiratory fitness, waist circumference, and body mass index). Anthropometric variables were measured at baseline and at 6, 12, 18, 24, 30 months, while cardiorespiratory fitness was measured at baseline, 6, 12, 18, and 30 months. The rationale for differences in the above measurements’ time points was to accommodate logistic challenges related to the original investigation. Reporting of this secondary analysis of the CHAMPS Study-DK follows the STROBE statement^23^.

*Physical activity* was measured using Actigraph GT3X accelerometers (Actigraph, Pensacola Florida). Trained research staff instructed children and parents on how to wear the device. Children wore the accelerometer on the right hip using a customized elastic belt from waking in the morning until they went to bed, except when bathing or swimming.

A customized software program (Propero, version 1.0.18, University of Southern Denmark, Odense, Denmark) processed all accelerometry data. Accelerations were recorded every 2 s and subsequently collapsed into 10-s epochs. Although, some data suggest shorter epochs length to quantify PA in children, a10-second epoch was selected based on the rationale that other data using shorter epochs length showed an overestimation of sedentary behaviour in children. Therefore, to optimize the accuracy of the sedentary data without compromising the vigorous PA data, we used the 10-s epochs. Digitalized accelerometer signals were filtered with 0.25–2.5 HZ band limits. This filter process is a mathematical weighting function that contribute to alter movement of low or high frequency above or below these limits and therefore help eliminating accelerations not associated with human movement (e.g., vibration). To distinguish inactivity from periods of non-wear, we interpreted readings of zero activity lasting at least 30 consecutive minutes as ‘accelerometer non-worn’. Although, other suggest length of 45–60 min, others have been suggesting 30 min of zero activity as non-wear time in children for more accuracy^24^. Therefore, we decided to use the 30 min to enhance accuracy. Data included in this study were limited to children who wore the accelerometer for at least 10 h per day on 4 or more days during each week of measurement. To represent overall PA, average counts per minute (CPM) were calculated by the following formula: (total counts/minutes of wear time). Proportion of the day spent in sedentary time (0–25 counts), light (26–573 counts), moderate (574–1002 counts), and vigorous (≥ 1003 counts) PA intensities were identified using pre-established and validated cut-points according to Evenson et al.^25^ As our data were collected in 2-s epochs, they were re-integrated in 10-s epochs and Evenson’s cut-points were scaled to ensure they mirrored the cut-points proposed by Evenson et al. All PA outcomes were averaged across the two measurement periods.

*Cardiorespiratory fitness* was measured with the Andersen test. This is an intermittent maximal indirect indoor running test developed for children and youth^26^. Briefly, children ran as fast as possible down a 20 m lane, touched behind the line with one hand, and turned and ran back in the opposite direction. After 15 s, children stopped immediately when hearing a whistle. Following 15 s of rest, the process repeated, with children attempting to cover the greatest distance possible. The test outcome was total distance run by each child in 10 min, and this was monitored by trained research staff. This test has good test–retest reliability and concurrent validity when compared with direct VO_2max_ testing in children^27^.

*Anthropometric measurements* including height and weight were measured with children barefoot, wearing light clothes, without hats or helmets, and with empty pockets. Height was measured to the nearest 0.5 cm with a portable stadiometer (SECA 214, Seca Corporation, Hanover, MD, USA), and weight to the nearest 0.1 kg using a calibrated Tanita BWB-800S digital scale (Tanita Corporation, Tokyo, Japan). Body mass index (BMI) was calculated as: weight (kg)/height (m)^2^ and categorized as normal weight, overweight, or obese according to International Obesity Task Force criteria^28^.

Waist circumference was measured to the nearest 0.5 cm with a tape measure placed at the level of the umbilicus following normal expiration. The measure was taken twice, and if differences greater than 1 cm were observed, a third measure was obtained. The mean of the two closest measurements was reported. Waist circumference outcomes were used to classify children as normal weight, overweight, or obese using sex and age-adjusted criteria^29^.

### Statistical analyses

The multi-trajectory modeling has been reported in detail elsewhere^20^. Briefly, we constructed a group-based multi-trajectory model that identified clusters of children who followed similar patterns of concurrent change (i.e., trajectories) in BMI, waist circumference, and aerobic fitness^30^. For the trajectory model, we excluded participants with less than three outcome measures. Otherwise, missing data were estimated with maximum likelihood estimation which results in asymptotically unbiased parameter estimates when data are missing at random^31^. We identified 3 health-related trajectory subgroups: (1) high fitness/normal weight’, (2) ‘moderate fitness /normal weight’, and (3) ‘low fitness/overweight-obese’.

Descriptive data were presented as mean ± SD and N (%) for categorical variables unless otherwise stated. We used multinomial logistic regression models with robust standard errors to investigate the associations between the PA variables (CPM, proportion of day spent in sedentary time, and light, moderate, and vigorous activity) and the three health-related trajectory subgroups. Results were reported as relative risk ratios (RRR) and 95% confidence intervals (CI). RRR were reported for every 25% time spent in sedentary behaviour; every 5% time spent in light and moderate PA, and every 2% time spent in vigorous PA and MVPA. All percentages (5%, 25%, and 2%) represent proportion of the day. CPM was reported per standard deviation unit (SD: 137.7). These numbers were arbitrary selected to enhance the interpretability of the analyses. Data management and statistical analyses were performed using STATA Version 15.0 software (StataCorp, College Station, TX, USA). A p ≤ 0.05 was considered significant for all inferential analyses.

### Ethics approval and consent to participate

The study was performed in accordance with the Declaration of Helsinki, approved by the Regional Scientific Ethical Committee of Southern Denmark (ID S-20080047), and registered on January 3, 2008 with the Danish Data Protection Agency (J.nr. 2008-41-2240). All children gave verbal assent and all parents provided written informed consent to participate before study enrollment.

## Results

**Figure 1:**
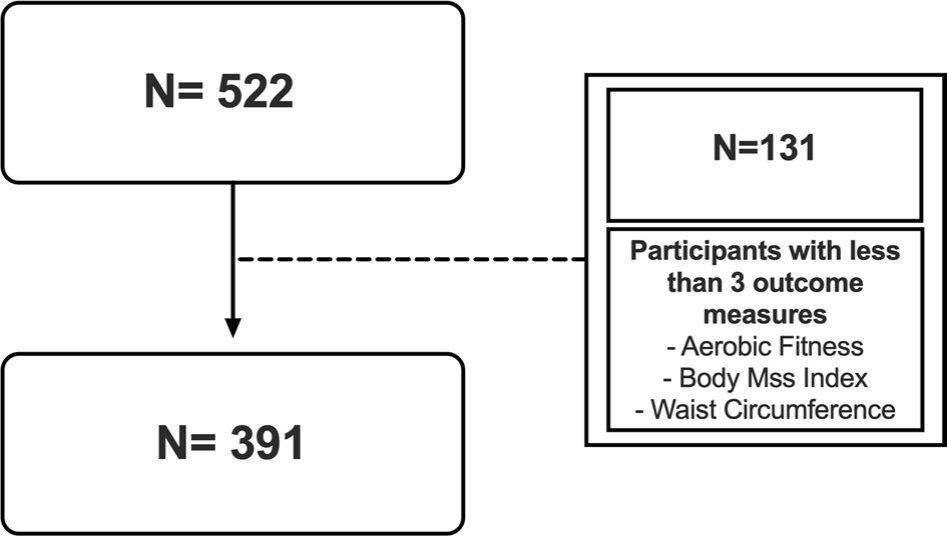
Flow chart of participants.

**Figure 2:**
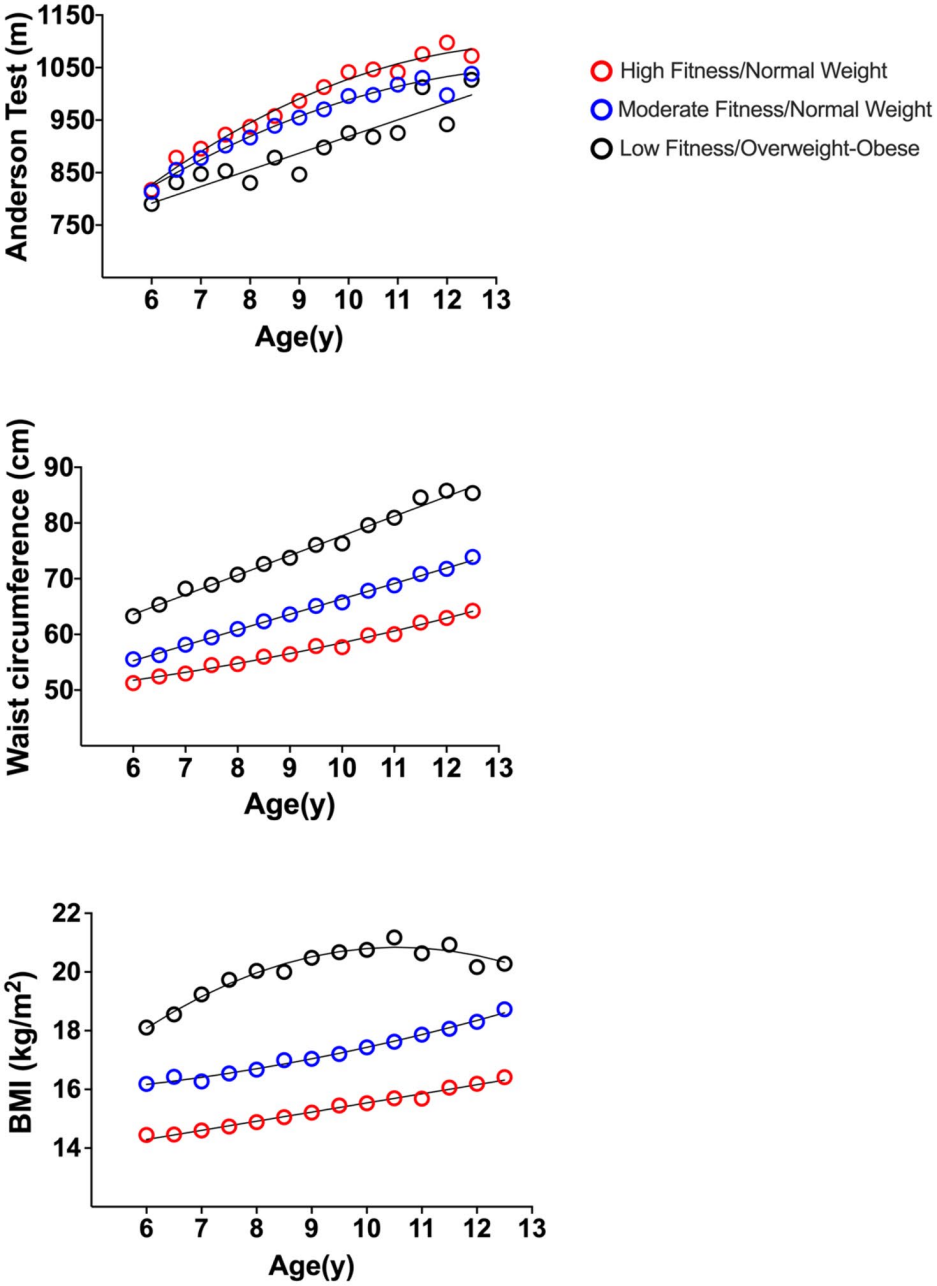
Developmental univariate trajectories of body mass index, waist circumference, and aerobic fitness for physically active children aged between 6 to 12.5 years.

In total, 522 children achieved 60 min of daily MVPA at each of the measurement periods. From this population, 131 children had less than 3 measures of BMI, waist circumference, or aerobic fitness. Therefore, data from 391 physically active children were included in the study analyses (Fig. 1). In total, 48.4% of children were classified as members of the ‘high fitness/normal weight’, 42.5% with ‘moderate fitness/normal weight’, and 9.1% with ‘low fitness/overweight-obese’ trajectory subgroups (Fig. 2).

Tables 1 and 2 describe the baseline characteristics of the sample and the PA and sedentary behaviour of the cohort as well as each trajectory subgroup. Briefly, physically active children were 8.1 ± 1.4 years old and 36.3% were girls. Mean accelerometer wear time was 797.5 ± 30.9 min/day. On average, children spent about 468.5 ± 45.3 min per day in sedentary behaviour, 50.4 ± 7.7 min in moderate PA, and 31.6 ± 8.3 min in vigorous PA.

**Table 1:** Descriptive baseline data stratified by health trajectory group.

Variables	Physically active children			
	Total sample (n = 391)	High fitness/normal weight (n = 187)	Moderate fitness/normal weight (n = 168)	Low fitness/overweight-obese (n = 36)
Age (years)	8.1 ± 1.4	8.2 ± 1.4	8.0 ± 1.3	8.0 ± 1.3
Female n (%)	142 (36.3)	58 (32.5)	62 (39.2)	14 (40.0)
Body mass index (kg/m^2^)	16.0 ± 1.6	14.9 ± 0.8	16.4 ± 0.9	19.3 ± 1.6
Overweight or obese n (%)	30 (8.1)	0 (0.0)	6 (3.7)	24 (68.5)
Waist circumferences (cm)	57.1 ± 5.5	55.6 ± 4.5	59.6 ± 4.7	67.4 ± 4.5
Andersen test (m)	909.9 ± 112.7	933.6 ± 110.3	901.9 ± 104.4	827. 3 ± 118.8

Data are presented as mean ± SD for continuous variables or n (%) for categorical variables.

**Table 2:** Physical Activity and Sedentary Behaviour Profile for the whole sample and according to based-group trajectory.

Variables	Physically active children			
	Total sample (n = 391)	High fitness/normal weight (n = 187)	Moderate fitness/normal weight (n = 168)	Low fitness/overweight-obese (n = 36)
Mean counts per minute	793.4 ± 137.8	812.2 ± 137.4	783.1 ± 140.4	744.4 ± 111.4
Mean minutes per day worn	797.5 ± 30.9	796.3 ± 30.6	799.0 ± 29.9	797.1 ± 32.4
Sedentary time (min/day)	468.5 ± 45.3	470.9 ± 45.5	467.3 ± 44.8	461.4 ± 74.0
Light PA (min/day)	246.7 ± 30.1	241.0 ± 28.0	250.4 ± 30.0	259.5 ± 35.1
Moderate PA (min/day)	50.4 ± 7.7	50.9 ± 7.8	50.2 ± 8.0	48.9 ± 6.0
Vigorous PA (min/day)	31.6 ± 8.3	33.1 ± 8.6	31.0 ± 8.0	27.1 ± 6.0
MVPA (min/day)	82.1 ± 13.0	84.1 ± 13.3	81.2 ± 12.9	76.0 ± 9.6

Data are presented as mean ± SD for continuous variables.

*PA* physical activity, *MVPA* moderate-to-vigorous physical activity.

**Figure 3:**
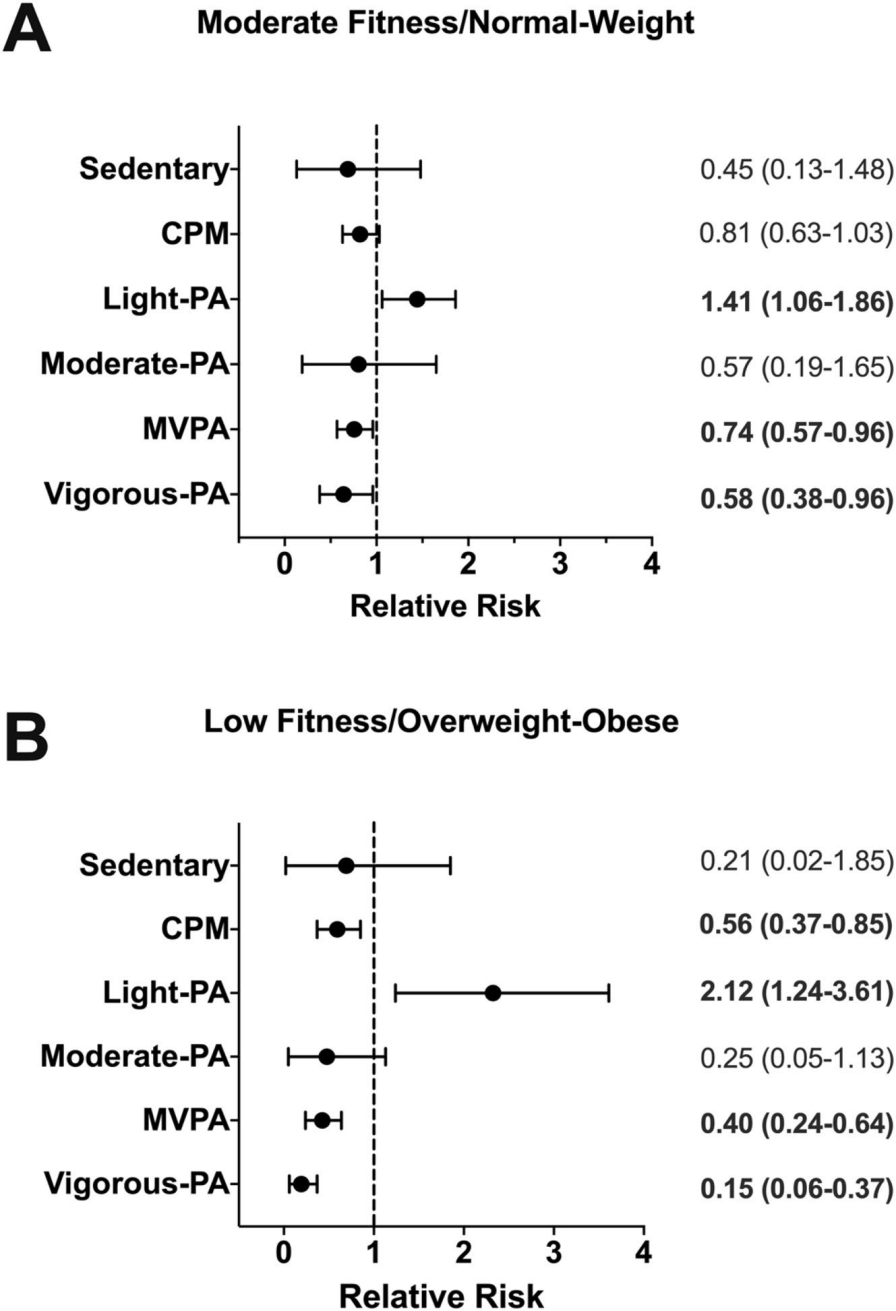
Relative risk ratios of being in the moderate fitness/ normal weight or in the low fitness/overweight-obese trajectory according to physical activity intensities. Data are presented as relative risk ratios (RRR) and (95% confidence intervals). RRR for sedentary behaviour is reported for every 25% time spent; light and moderate PA is reported for every 5% time spent, while vigorous PA and MVPA are reported for every 2% time spent at that intensity, CPM is reported per standard deviation (SD:137.7496); Reference group is children assigned to the ‘high fitness/normal weight’ trajectory.

Figure 3 reports the associations between sedentary behaviour and PA and the health-related trajectory subgroups. There were no associations between total sedentary behaviour or time in moderate intensity physical activity and trajectory group membership (Fig. 3A,B). Children who spent more time in light intensity PA were more likely to be classified as members of the ‘moderate fitness/ normal weight’ [RRR (95% CI) = 1.41 (1.06 to 1.86) per 5% increase] and ‘low fitness/ overweight-obese’ [RRR (95% CI) = 2.12 (1.24 to 3.61) per 5% increase] subgroups than children following a ‘high fitness/normal weight’ trajectory. Conversely, children who spent more time in MVPA were less likely to be classified as members of the ‘moderate fitness/normal weight’ [RRR (95% CI) = 0.74 (0.57 to 0.96) per 5% increase] and ‘low fitness/overweight-obese’ [RRR (95% CI) 0.40 (0.24–0.64) per 5% increase] subgroups than children following a ‘high fitness/normal weight’ subgroup. Children who spent more time in vigorous PA were less likely to be classified as members of the ‘moderate fitness/normal weight’ subgroup [RRR (95% CI) = 0.58 (0.38 to 0.96) per 2% increase] and ‘low fitness/overweight-obese’ [RRR (95% CI) 0.15 (0.06–0.37) per 2% increase] subgroups compared to ‘high fitness/normal weight’. Children with more CPM were less likely to be classified as members of the ‘Low Fitness/ Overweight-Obese’ [RRR (95% CI) = 0.56 (0.37 to 0.85) per 1SD increase in CPM compared to ‘high fitness/normal weight’.

## Discussion

The aim of this study was to investigate the prospective associations between PA behaviour (overall PA and PA intensity) and trajectories of health-related factors in children meeting PA guideline recommendations. Among PA guideline concordant children, PA behaviours were associated with membership in the health-related trajectory subgroups. Children who performed more PA overall were less likely to be classified as members of the unfavorable health trajectory. This result aligns closely with the world health organization guideline on PA and sedentary behaviour^32^ suggesting that every step counts for overall health. In addition, our results suggest that children who spent more time in MVPA and vigorous PA were less likely to be classified as members of the unfavorable health trajectory. Conversely, children who spent more time engaged in light intensity PA were more likely to follow an unfavourable health trajectory. These results suggest that (1) a small increase in overall PA or (2) even a small increase in PA intensity in children who adhere to the national recommendation of PA may have important health benefits. For example, 8 min of MVPA and 6 min of vigorous PA appears beneficial to reduce the risk of following an unfavourable health trajectory.

The main finding of this study is the strong association between the reduced risk of being in the ‘low fitness/overweight-obese’ trajectory and time spent in MVPA, particularly vigorous PA. In a cross-sectional study of 29,734 children, Tarp et al., investigated PA patterns in children and found that intensity rather than duration was the strongest predictor of a reduction in BMI^33^. These results highlighted that in children PA should be promoted especially at higher intensities. Similarly, results from a cross-sectional study performed by Hay et al. showed a 44% reduction in the likelihood of being overweight for every 7 min of vigorous PA^34^. Finally, a meta-analysis of cross-sectional studies investigated the reallocation of sedentary time to MVPA and observed a reduction in the percent body fat, although a reduction in BMI and waist circumference was not observed^35^. The results of our study are important as they provide longitudinal evidence that a small addition of vigorous PA of about 6 min, appears to reduce the risk of following an unhealthy trajectory in children meeting the national recommendation of PA. The current study adds to the whole body of knowledge on exercise response by providing prospective evidence that vigorous PA predicts developmental health trajectories in children meeting PA guideline recommendations.

PA mainly reduces chronic disease risk by reducing adiposity and increasing cardiorespiratory fitness^19^. Based on our results, vigorous PA is the strongest predictor of such improvements. Vigorous PA confers beneficial physiological adaptations that increase cardiorespiratory fitness and reduce adiposity and waist circumference. For example, vigorous PA may increase cardiorespiratory fitness through enhanced oxygen extraction capacity in the skeletal muscles^36^. Furthermore, vigorous PA is associated with a greater release of catecholamines compared to lighter PA, which activate adipose tissue lipolysis via hormone sensitive lipase, especially in the abdominal area^37^. Therefore, these mechanisms may explain some of the improvements in adiposity and waist circumference associated with vigorous PA.

In the current study, we observed that PA guideline concordant children demonstrate measurable inter-individual variation in their development of BMI, waist circumference, and aerobic fitness. This aligns with results from other studies performed in children, suggesting that although many children benefit from PA interventions, some children do not experience health-related benefits^13–16^. For example, A prospective 6 month study involving 79 sedentary children at risk of Type 2 diabetes, reported large heterogeneity in cardiorespiratory fitness and adiposity following a PA program^13^. Despite implementing well-controlled procedures, the inter-individual variation in several adiposity measures ranged from a decrease of 69% to an increase of 55%^13^. Results from the current study support the concept of heterogeneity in exercise response in children, and build on our previous findings by virtue of including a large cohort of children meeting the national PA recommendations.

Although this study revealed important findings, several limitations need to be discussed. First, the relatively small number of children in the ‘low fitness/overweight-obese’ trajectory did not allow us to adjust for multiple covariates simultaneously including socioeconomic status and education. Second, accelerometers worn at the hip level do not capture movements such as cycling or swimming which may have impacted our results. Third, habitual PA was not quantified throughout the study, which would have informed us on participant’s PA level between the two time-point measurement, which could have impacted our results. Fourth, no information in terms of diet quality and energy intake were available and we did not account for weekend time PA which might contribute the explanation of our results. Finally, since only about a third of our sample was composed of girls, our data are more representative of active boys. Despite these limitations, our study is strengthened by the prospective design, the relatively large sample size and objective measure of PA. In addition, using a novel multi-trajectory approach, our study used multiple concurrent health outcomes to explore PA predictors in active children.

## Conclusion

In conclusion, our results suggest that amongst physically active children, those children belonging to the low aerobic fitness and overweight-obese trajectory had lower overall PA, spend less time in MVPA and more time in light-intensity PA. In particular, the amount of vigorous PA was associated with better health trajectories in these children and was the strongest predictor of maintaining a better health trajectory. Globally, PA guidelines should consider the importance of vigorous PA as part of their daily PA in children.

## Data Availability

Data are available from the CHAMPS Study Steering Committee upon reasonable request. Legal and ethical restrictions apply. Interested parties may contact Dr. Niels Christian Møller (nmoller@health.sdu.dk), and the following information will be required at the time of application: a description of how the data will be used, securely managed, and permanently deleted.
